# Investigation of Gasochromic Rhodium Complexes Towards Their Reactivity to CO and Integration into an Optical Gas Sensor for Fire Gas Detection

**DOI:** 10.3390/s18071994

**Published:** 2018-06-21

**Authors:** Carolin Pannek, Karina R. Tarantik, Katrin Schmitt, Jürgen Wöllenstein

**Affiliations:** 1 Fraunhofer-Institute for Physical Measurement Techniques IPM, Heidenhofstrasse 8, 79110 Freiburg, Germany; karina.tarantik@ipm.fraunhofer.de (K.R.T.); katrin.schmitt@ipm.fraunhofer.de (K.S.); juergen.woellenstein@ipm.fraunhofer.de (J.W.); 2 Laboratory for Gas Sensors, Department of Microsystems Engineering—IMTEK, University of Freiburg, Georges-Köhler-Allee 102, 79110 Freiburg, Germany

**Keywords:** gasochromic color dye, rhodium complexes, carbon monoxide, gas sensor

## Abstract

The detection of the toxic gas carbon monoxide (CO) in the low ppm range is required in different applications. We present a study of the reactivity of different gasochromic rhodium complexes towards the toxic gas carbon monoxide (CO). Therefore, variations of binuclear rhodium complexes with different ligands were prepared. They were characterized by FTIR spectroscopy, ^1^H NMR spectroscopy, and differential scanning calorimetry. All complexes are spectroscopically distinguishable and temperature stable up to at least 187 °C. The gasochromic behavior of all different compounds was tested. Therefore, the compounds were dissolved in toluene and exposed to 100 ppm CO for 10 min to investigate their gas sensitivity and reaction velocity. The changes in the transmission spectra were recorded by UV/vis spectroscopy. Furthermore, a significant influence of the solvent to the color dyes’ gasochromic reaction and behavior was observed. After characterization, one complex was transferred as sensing element into an optical gas sensor. Two different measurement principles (reflection- and waveguide-based) were built up and tested towards their capability as gasochromic CO sensors. Finally, different gas-dependent measurements were carried out.

## 1. Introduction

Carbon monoxide (CO) is a toxic gas, which occurs by incomplete combustion of organic substances, like coal or crude oil. Since it is colorless, odorless, and toxic at low concentration levels, it is difficult to detect. The intoxication by carbon monoxide is a frequent cause of human injury. Especially in Europe, it is the most common poisoning in case of home fires with fatal outcome [1]. About 80–90% of affected people die due to gas exposure and not by flames, because, in general, there is only 4 min of time to escape. CO evolves not only in fires, but also in damaged exhaust air units, heating systems, and gas furnaces. This may result in a distribution of the toxic gas in workspaces and living areas causing physical injuries.

For these reasons, there is an immense interest in the development of sensor systems capable of detecting carbon monoxide at low concentrations. Conventional home-use fire detectors cannot measure gas concentrations, but detect all sorts of particles in air. In case of a fire, the light of a LED scatters at fume particles, and induces a signal at a photo detector. Exceeding a critical value causes the fire alarm. The principle is based on the assumption that all fire types produce fume particles [2]. These light-scattering detectors are low-priced (<20 €), and work for up to ten years, but they have a number of disadvantages. Next to fires, other particle producing events, like cooking or taking a hot shower, can cause alarms. To detect fires with higher probability, it is important to detect typical fire gases like CO [3]. Requirements for commercial fire detectors include low-cost (10 €) and low-power <1 mW, so that they work with a battery for up to ten years.

One attempt to improve the gas detecting properties of fire detectors is to integrate a chemical gas sensor next to the common light-scattering sensor [4]. Commercially available sensors for quantitative CO detection in air are, up to now, limited to semi-conductive (MOS or GasFETs) or electrochemical sensors. These sensors have different disadvantages, which complicates their usage. Palladium-doped SnO_2_ sensors are very sensitive to CO [5], but suffer from high incidence of false alarms, due to their cross sensitivities to water and all reducing and oxidizing gases. Furthermore, they have working temperatures from 150–400 °C, with power consumption far higher than 100 mW. This implies that two AA mignon batteries have a lifespan of a few days, which makes battery operation impossible. Modern approaches, using hotplate structures or temperature pulsed modes, might promote the integration of MOS [6]. Nevertheless, a battery mode for up to ten years is impossible by now.

Compared to MOS, GasFETs work at room temperature and have clearly lower power consumption <1 mW, which would be ideal for the here presented application [7]. Different GasFETs with different gas sensing layers could detect fire typical gases, like CO, NO_2_, and CO_2_, and can even discriminate between smoldering and open fires by using a sensor array and signal processing [7]. In this case, the disadvantages are high costs and increased power consumption for intelligent signal processing.

More selective chemical sensors are electrochemical cells (ECs). Most electrolytes react to only one target gas. The gas reaction consumes the electrolyte that limits the expected lifetime to about 36 months, which is not suitable for fire detectors. Furthermore, ECs suffer from higher costs [8].

These facts underline the importance of alternative sensing methods for fire detectors. Our approach is based on the optical detection of gas sensitive color dyes. Colorimetric sensor systems are gaining increasing significance as an alternative technique for the detection of CO. They offer various advantages, such as quick response time, high selectivity, and the use of simple instrumentation.

Gas sensitive color dyes have been chemically known about for many years. An overview of gasochromic color dyes for CO detection is given in [9]. The binuclear rhodium complex used for this work was firstly described by Esteban in 2010 [10]. Binuclear rhodium complexes show a fast and selective reaction towards CO [10,11,12]. Furthermore, these compounds offer the advantage of a reversible CO reaction. Therefore, we prepared several binuclear rhodium complexes starting from the dimer rhodium(II) acetate. For evaluation of the reaction velocity and sensitivity towards CO, the color change of rhodium complexes, dissolved in different solvents, while applying CO, was recorded.

To create the path from the sensitive material to a sensor system, we developed a waveguide- and reflection-based sensor setup which is capable of detecting carbon monoxide at low concentration levels in air, and is suitable for low-priced mass production [13].

## 2. Sensing Principles

We investigated two different sensing principles to detect the color change of gasochromic material. The first approaches are reflection-based measurements, the second, waveguide-based.

### 2.1. Reflection-Based Measurements

**Figure 1:**
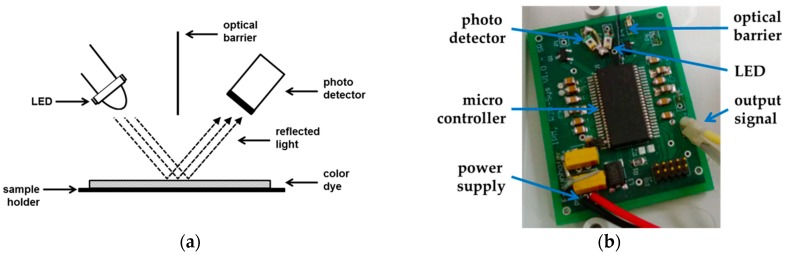
(**a**) Sketch of the measuring principle. The color change of the sensitive probe can be detected by the reflected light of a blue LED on the surface of the color dye. To avoid interfering light, an optical barrier is mounted between the LED and the detector; (**b**) Picture of the realized measurement system. The measuring board is fixed towards the sensitive probe.

The working principle of the reflection-based system is based on the optical modulations of colored silica in the presence of CO. To detect a maximum signal shift, the LED has to fit to the spectral range of the color change. Therefore, a low-power and low-cost measurement setup, mainly consisting of a blue emitting LED, a photo detector and the CO-sensitized silica gel, was configured. The main emitting wavelength of the LED is 470 nm, which is fitting to the main sensitive region of the color dye towards CO. The photo detector measures the reflected light intensity of the colored silica gel. For maximum signal change a barrier between the LED and the photo detector prevents direct irradiation on the detector, as shown in Figure 1.

### 2.2. Waveguide-Based Measurements

**Figure 2:**
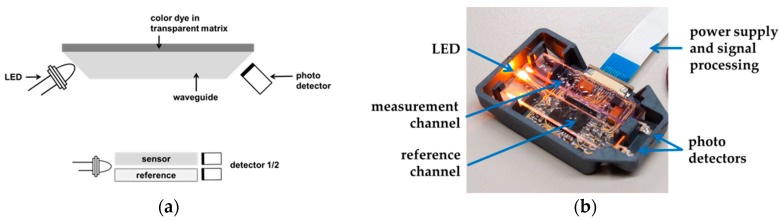
(**a**) Sketch of the gasochromic sensor, consisting of a planar optical waveguide, covered with the colorimetric film. The light of an LED is coupled into one end of the waveguide, and travels through it under TIR before it is focused onto a photo detector. The gas reaction leads to changes in the detector signal. Top view of the sensor element consisting of the measurement channel, a reference channel, two photo detectors, and one LED; (**b**) Picture of the waveguide-based measurement system realized by R2R processing [13].

One opportunity to improve the sensitivity of a gasochromic sensor system is to perform waveguide-based measurements. In this setup, the sensitive layer is deposited on top of an optical waveguide. The light of a LED is coupled into the front side of the waveguide, and passes through it under the conditions of total internal reflection (TIR). The color change can be detected as absorption changes inside the layer. After coupling out, the light is focused on a photo detector. Changes in the dye induce direct changes in the intensity of the light. Compared to transmission-based measurements, the waveguide principle is more sensitive due to a longer optical path. This measurement principle has already been described in [14,15]. To be independent from environmental influences, a reference channel is embedded additionally. The final sensor setup is shown in Figure 2.

## 3. Experimental

### 3.1. Variation of Binuclear Rhodium Complexes

**Figure 3:**
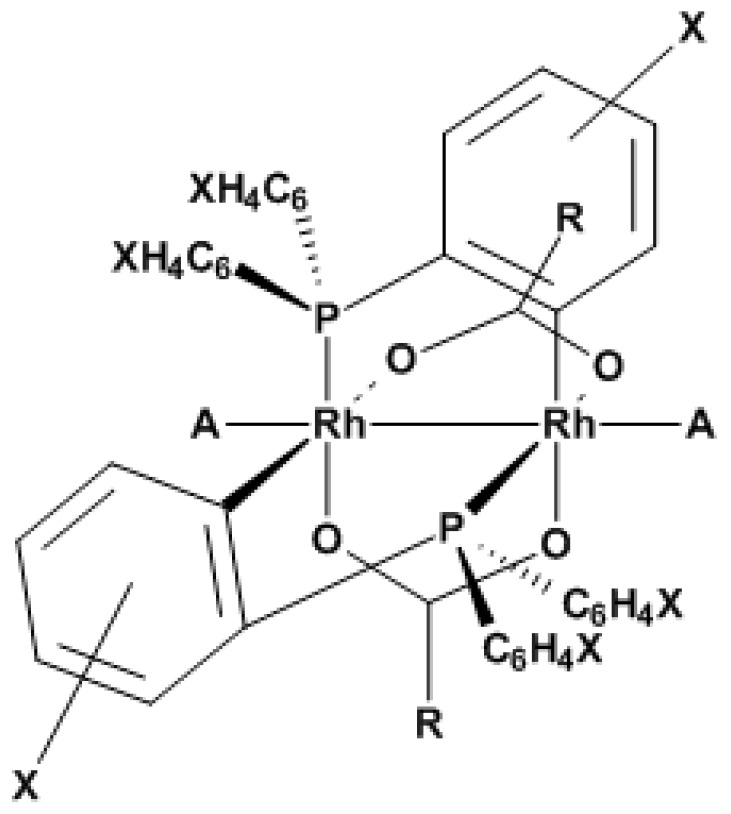
Chemical structure of the gasochromic binuclear rhodium complex [Rh_2_{(XC_6_H_3_)P(XC_6_H_4_)}_n_(OCR)_2_]∙**A_2_** with different ligands (see Table 1).

As an indicator for CO, binuclear rhodium complexes were investigated. They consist of the structure [Rh_2_{(XC_6_H_3_)P(XC_6_H_4_)}n(OCR)_2_]∙**A_2_**, shown in Figure 3. Two rhodium ions form the linear center of the binuclear complex. They are linked with two carboxyl groups and two triphenylphosphines. The ligands **A** in axial direction are simply coordinated to the central metal atoms. The complexes were synthesized according to a procedure described in the literature [11,12,16]. By varying the residues of the phosphine and carboxyl group, five different compounds were obtained (see Figure 3 and Table 1). For modification of the phosphines, different residues **X** of the phosphine ligands with electron-donative properties, like methyl (–CH_3_) or methoxy (–OCH_3_), and electron-withdrawing, like fluorine (–F), were used.

For synthesis, 0.45 mmol (200 mg) rhodium(II) acetate dimer were dissolved in a mixture of 25 mL toluene and 5 mL acetic acid, and 0.90 mmol of the corresponding phosphine were added. After refluxing the mixture at 125 °C for 3 h, the solution was cooled to ambient temperature. The solvent was removed under reduced pressure at ambient temperature to yield a deep purple (**1∙(A)_2_**–**3∙(A)_2_**, **5∙(A)_2_**) or green powder (**4∙(A)_2_**). The corresponding phosphines are tris(4-methoxyphenyl)phosphine for **1∙(A)_2_**, tris(3-methylphenyl)phosphine for **2∙(A)_2_** and **5∙(A)_2_**, tris(3-fluorophenyl)phosphine for **3∙(A)_2_**, and tris(4-methoxy-3,5-dimethylphenyl)phosphine for **4∙(A)_2_**. Besides the four complexes with acetic acid as ligand **A**, a complex with trifluoroacetic acid (**5∙(A)_2_**) was prepared by dissolving 100 mg **2∙(A)_2_** in 5 mL toluene. After adding 2 mL trifluoric acid, the solution was stirred at room temperature for 10 min. By evaporation of the solvent, a purple powder was obtained, and recrystallized in CHCl_3_.

The rhodium complexes were characterized by FTIR spectroscopy (Bruker ALPHA-T), ^1^H NMR spectroscopy (Bruker Avance 250, Bruker, Billerica, MA, USA) and differential scanning calorimetry (Netzsch DSC 204 F1, Netzsch, Selb, Germany); heat rate 10 K/min).

**1∙(A)_2_**: Yield: 80%. FTIR (KBr, cm^−1^): 3432 (w), 3064 (vw), 2995 (w), 2935 (m), 2903 (w), 2834 (m), 2618 (vw), 2543 (w), 2050 (w), 1893 (w), 1678 (s), 1592 (vs), 1566 (vs), 1502 (vs), 1485 (s), 1429 (s), 1404 (s), 1366 (m), 1342 (m), 1287 (vs), 1249 (vs), 1218 (s), 1179 (vs), 1121 (m), 1096 (s), 1028 (s), 941 (w), 862 (w), 825 (s), 796 (s), 717 (w), 689 (m), 619 (m), 598 (w), 547 (s), 501 (m), 466 (m), 432 (m). ^1^H NMR (250 MHz, CDCl_3_, 300 K): δ (ppm) = 1.29 (s, 6 H), 1.34 (s), 2.14 (s, 6 H), 3.76 (s, 6 H), 3.78 (s), 3.80 (s, 6 H), 3.84 (s), 3.94 (s), 6.65 (m, 16 H), 7.58 (m, 6 H). DSC: 187 °C (dec).

**2∙(A)_2_**: Yield: 92%. FTIR (KBr, cm^−1^): 3432 (w), 3050 (vw), 3009 (w), 2918 (m), 2862 (w), 2618 (vw), 2548 (w), 2355 (vw), 1678 (vs), 1569 (s), 1474 (m), 1446 (s), 1410 (s), 1367 (m), 1335 (m), 1294 (s), 1243 (m), 1219 (vw), 1172 (w), 1138 (w), 1107 (m), 1083 (w), 1024 (m), 994 (vw), 940 (vw), 869 (w), 815 (m), 777 (m), 724 (w), 693 (s), 631 (vw), 612 (vw), 558 (m), 530 (w), 469 (s), 431 (vw). ^1^H NMR (250 MHz, CDCl_3_, 300 K): δ (ppm) = 1.25 (s, 6 H), 1.93 (s, 6 H), 2.06 (s, 6 H), 2.16 (s, 6 H), 2.36 (s, 6 H), 6.33 (m, 6 H), 7.10 (m, 12 H), 7.35 (m, 4 H). DSC: 214 °C (dec).

**3∙(A)_2_**: Yield: 97%. FTIR (KBr, cm^−1^): 3432 (w, b), 3064 (w), 2927 (w, b), 2678 (vw), 2620 (w, b), 2554 (w, b), 2358 (vw), 1671 (vs), 1600 (m), 1576, (vs), 1475 (s), 1417 (vs), 1364 (m), 1342 (m), 1295 (m), 1262 (m), 1244 (s), 1217 (vs), 1186 (m), 1164 (w), 1138 (vw), 1117 (w), 1096 (m), 1067 (w), 1046 (vw), 1024 (m), 997 (w), 893 (m), 876 (m), 820 (w), 783 (m), 720 (w), 687 (vs), 631 (vw), 614 (w), 591 (m), 573 (m), 523 (m), 493 (s), 453 (w), 414 (w). ^1^H NMR (250 MHz, CDCl_3_, 300 K): δ (ppm) = 1.36 (s, 6 H), 2.20 (s, 6 H), 6.39 (m, 2 H). 6.56 (m, 4 H), 7.01 (m, 8 H), 7.40 (m, 8 H). DSC: 231 °C (dec).

**4∙(A)_2_**: Yield: 37%. FTIR (KBr, cm^–1^) = 3436 (w, b), 2928 (m), 2859 (m), 2825 (m), 2731 (vw), 2004 (vw), 1922 (vw), 1759 (vw), 1563 (s), 1478 (s), 1449 (m, sh), 1419 (vs), 1399 (s), 1340 (w), 1279 (s), 1260 (m), 1221 (s), 1177 (w), 1143 (m), 1115 (vs), 1013 (vs), 955 (vw), 908 (m), 875 (m), 802 (m), 763 (w), 731 (vw), 684 (m), 658 (vw), 618 (s), 579 (w), 548 (m), 510 (m), 464 (w), 429 (vw). ^1^H NMR (250 MHz, CDCl_3_, 300 K): δ (ppm) = 1.45 (s), 1.50 (s), 1.98 (s), 2.07 (s), 2.20 (s), 2.27 (s, 6 H), 2.31 (s), 6.38 (m, 2 H), 7.24 (m, 8 H). DSC: 228 °C (dec).

**5∙(A)_2_**: Yield: 81%. FTIR (KBr, cm^–1^) = 3422 (w, b), 3053 (vw), 3019 (vw), 2919 (w), 2866 (vw), 2361 (vw), 1652 (s), 1619 (s), 1595 (w), 1536 (vw), 1479 (w), 1446 (m), 1370 (vw), 1243 (w), 1201 (vs), 1152 (s), 1105 (m), 1090 (w), 1024 (m), 995 (vw), 856 (m), 818 (w), 780 (m), 733 (s), 696 (m), 562 (m), 526 (w), 476 (s), 432 (vw). ^1^H NMR (250 MHz, CD_2_Cl_2_, 300 K): δ (ppm) = 2.02 (s, 6 H), 2.12 (s, 6 H), 2.39 (s, 6 H), 6.40 (m, 6 H), 7.29 (m, 16 H). DSC: 215 °C (dec).

### 3.2. Sample Preparation

The sensing probe of our reflection-based system is achieved by the adsorption of **2∙(A)_2_** compound on fine silica. Therefore, the silica and the rhodium complex were mixed in chloroform. During the drying step, the chloroform vaporizes, and the rhodium complex adsorbs on the silica surface.

For all waveguide-based measurements, the color dye was embedded into a polymer matrix. This matrix has to fulfill the following requirements: high porosity for diffusion of the target gases, inert to environmental influences, like humidity, temperature, and radiation, inert to the colorimetric material, and ideally the same solvent as the dye. Possible polymers are polyvinyl chloride (PVC), polydimethylsiloxane (PDMS), ethyl cellulose (EC), and/or poly(methyl methacrylate) (PMMA). Each dye/polymer combination is unique, and has to be determined individually. The liquid sensitive matrix can be deposited onto the waveguide by dip- or spin-coating.

The following results were achieved using **2∙(A)_2_** embedded into an EC matrix. The polymer solution consists of 1 g EC powder and 1 mL tributyl phosphate (both Sigma Aldrich, Darmstadt, Germany) as plasticizer. Both were dissolved in 40 mL ethanol (pure grade) and stirred for 2 h under ambient conditions. Finally, **2∙(A)_2_** was added in a concentration of 6.6 mg/L. For the sensor chips, 100 µL **2∙(A)_2_**/EC solution was pipetted on the optical waveguide and dried for 1 h at 50 °C. This results in a layer thickness of 2500 nm.

### 3.3. Gas-Dependent Measurements

All measurements were performed at the gas measurement station of Fraunhofer IPM. It comprises of calibrated mass flow controllers to provide a defined and stable gas flow of a mixture of up to ten gases. The humidity is variable, between 0% r.H and 80% r.H., by passing nitrogen through a temperature-controlled dual flask bubbler filled with deionized water. To provide constant ambient conditions, the measurements systems are installed in a climatic chamber (Vötsch VT 7004, Vötsch Industrietechnik, Reiskirchen, Germany). The general description of the gas measurement station is given in [17].

## 4. Results

### 4.1. Colorimetric Reaction

**Figure 4:**
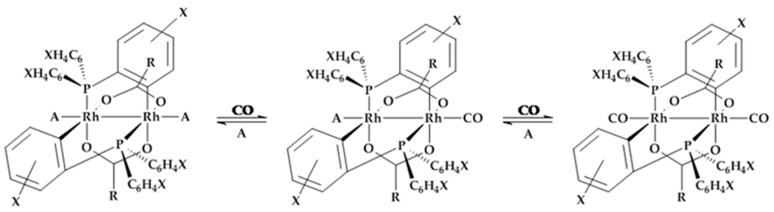
Principal reaction scheme of the rhodium complex with CO. The reaction induces a two stages ligand exchange of **A** with CO. Left: [Rh_2_{(XC_6_H_3_)P(XC_6_H_4_)}_n_(OCR)_2_]∙**(A)_2_**, Middle: [Rh_2_{(XC_6_H_3_)P(XC_6_H_4_)}_n_(OCR)_2_]∙**(A,CO),** Right: [Rh_2_{(XC_6_H_3_)P(XC_6_H_4_)}_n_(OCR)_2_]∙**(CO)_2_**.

The color change of the prepared rhodium complexes is caused by a reversible two-step ligand exchange depicted in Figure 4. Each complex molecule can react independently with two CO molecules by ligand exchange of **A** in axial direction. This two-step mechanism causes a color change over different color nuances. Variations in the reaction time can be explained by the more or less electron-donating residues **X** of the phosphine ligands. Thereby, compound **2∙(A)_2_** showed the fastest, and compound **3∙(A)_2_** the slowest reactivity with CO. To obtain a reversible reaction, the substituted ligand **A** remains near the complex by hydrogen bridge bonds to the carboxyl groups. All complexes show the reverse color reaction after stopping CO exposure. The equilibrium has its preferred reaction towards the CO exchange, which offers faster forward reactions.

**Figure 5:**
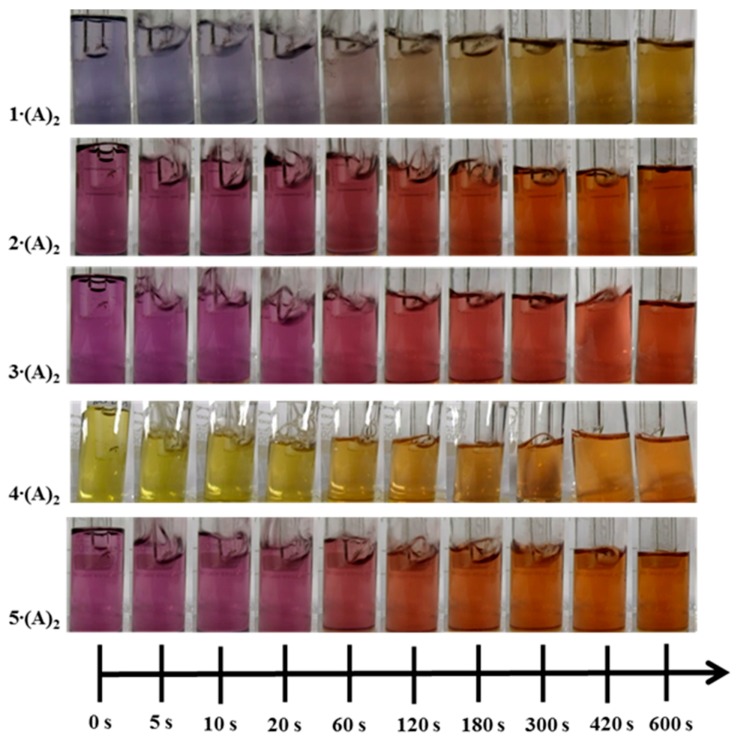
Color changes of the rhodium complexes **1∙(A)_2_**–**5∙(A)_2_** dissolved in toluene, during the exposure to 100 ppm CO (40% r.H.) for 10 min.

**Figure 6:**
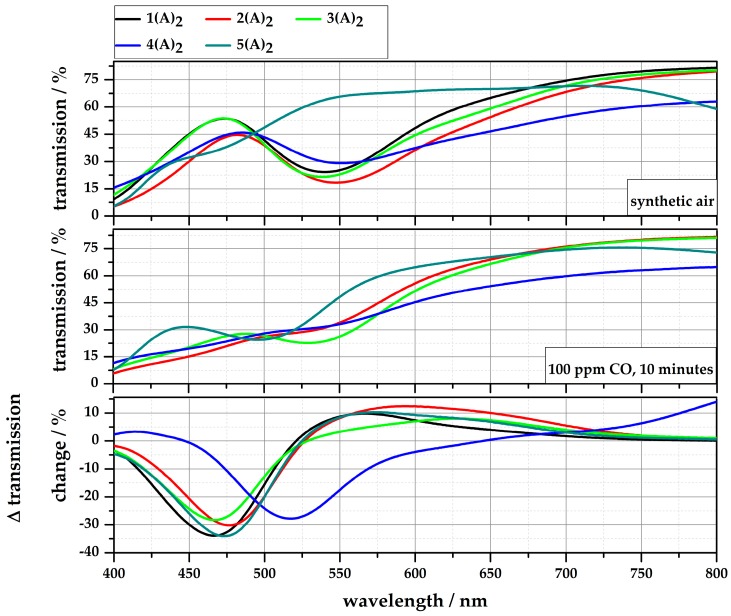
Difference of the transmission change (ΔT/%) of the investigated Rh compounds before and after exposure to 100 ppm for 10 min.

Upon CO exposure, the rhodium complexes change theirs color from purple through red and orange to yellow (**1∙(A)_2_**–**3∙(A)_2_**, **5∙(A)_2_**) or from light green to orange (**4∙(A)_2_**). The gradient of the color change of the solutions due to an exposure of 100 ppm CO (40% r.H.) for 10 min is shown in Figure 5. For all complexes, the color change is recognizable after 20 s by the naked eye. To obtain quantitative information of the color gradient, the UV/vis spectra of each solution was recorded before and after the exposure to 100 ppm CO for 10 min. The measurements were performed in transmission between 350 nm and 800 nm. For easy comparison of the solutions, the difference in the transmission change (ΔT/%) is shown in Figure 6. The purple complexes (**1∙(A)_2_**–**3∙(A)_2_**, **5∙(A)_2_**) show their maximum transmission change in the blue wavelength area (~470 nm), the green one (**4∙(A)_2_**) in the green wavelength area (~517 nm). The detailed transmission changes and corresponding wavelengths are given in Table 2.

During the measurements, an influence of the solvent on the color and gas sensitivity of the complexes was observed. To determine this influence, **1∙(A)_2_** was solved in five different solvents: ethanol, propane-1,2-diol, toluene, chloroform, and water. Variations in the solubility and color intensity were directly identifiable. The complex was easily dissolved in ethanol, toluene, and chloroform to obtain clear violet solutions, while the complex is completely insoluble in water. The solution with propane-1,2-diol needed to be stirred for 30 min at room temperature to obtain a clear, but blue solution.

**Figure 7:**
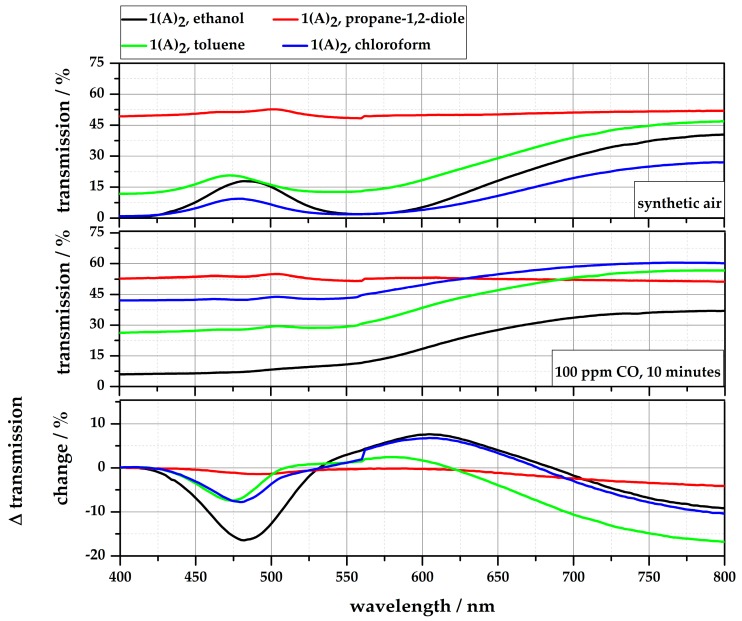
Difference of the transmission change (ΔT/%) of **1∙(A)_2_** dissolved in ethanol, propane-1,2-diole, toluene, and chloroform, before and after exposure to 100 ppm for 10 min.

As described, these solutions were exposed to 100 ppm CO for 10 min (40% r.H.) and the transmission spectra were recorded before and after gas exposure (see Figure 7). The results show an obvious influence of the solvent on the gas reaction. Ethanol leads to the highest transmission change of 16.4% at 481 nm, while the solution with propane-1,2-diol shows no significant color change. Additionally, the reaction time of the ethanol solution is the fastest compared to the other solvents. However, ethanol has the major disadvantage, in that the solution is not stable over time (>12 weeks). Gradually, a ligand exchange between **A** and ethanol occurs, so that the solution also turns yellow. The transmission changes of the solutions with toluene and chloroform are comparable at about 480 nm, only toluene shows lower transmission changes in the orange range (~580 nm). An overview of the properties of dissolved **1∙(A)_2_** regarding solubility, color, stability, and gas sensitivity is given in Table 3. The quantitative evaluation is based on the results of the measurements in Figure 6 and Figure 7.

### 4.2. Reflection-Based Measurements

**Figure 8:**
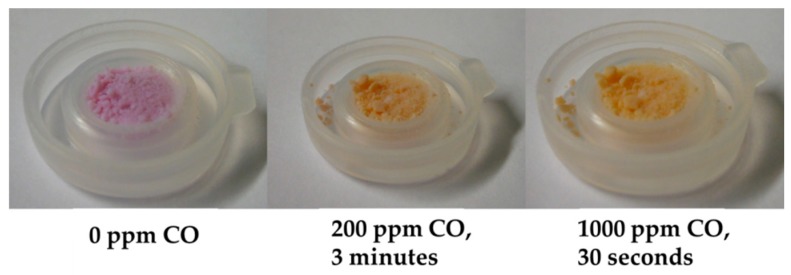
Color change of **1∙(A)_2_** adsorbed on silica gel before (left) and after exposure to 50–1000 ppm CO in synthetic air at 50% r.H. Each picture was taken after 30 s of CO exposure. After CO exposure, the color change is reversible, and the silica reaches its original color again.

**Figure 9:**
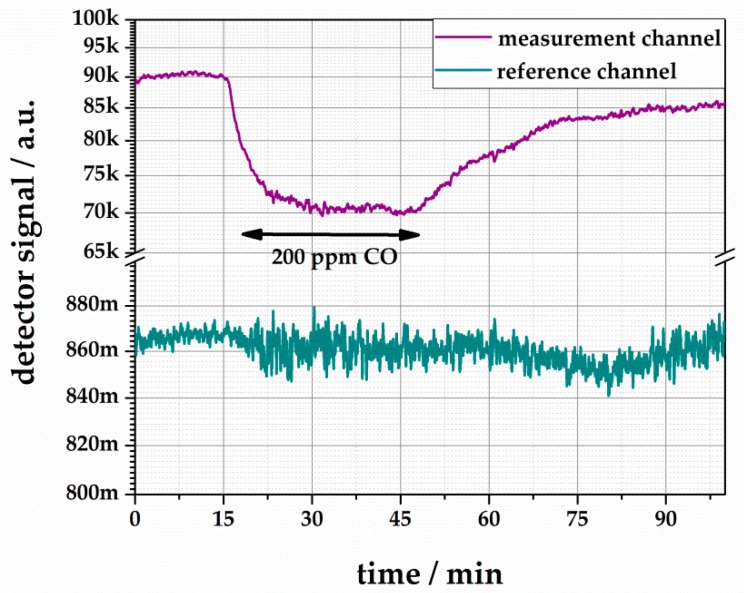
Colorimetric reaction of **1∙(A)_2_** adsorbed on silica gel to 100 ppm CO with 40% r.H. at room temperature.

The colored silica undergoes a fast and defined color change within seconds from purple over orange to yellow when it is exposed to carbon monoxide in air. Pictures of the colored silica and its color change are shown in Figure 8. An according gas measurement, using our measurement system (see Figure 1b), is shown in Figure 9. The sensor was exposed to 200 ppm CO, and its response is divided into the signals of the measurement and the reference channel. Due to the diffuse surface of the silica particle, the signals show significant noise. The response time can be determined as T_10_ = 80 s. The measurement also shows the reversible behavior of the color dye.

**Figure 10:**
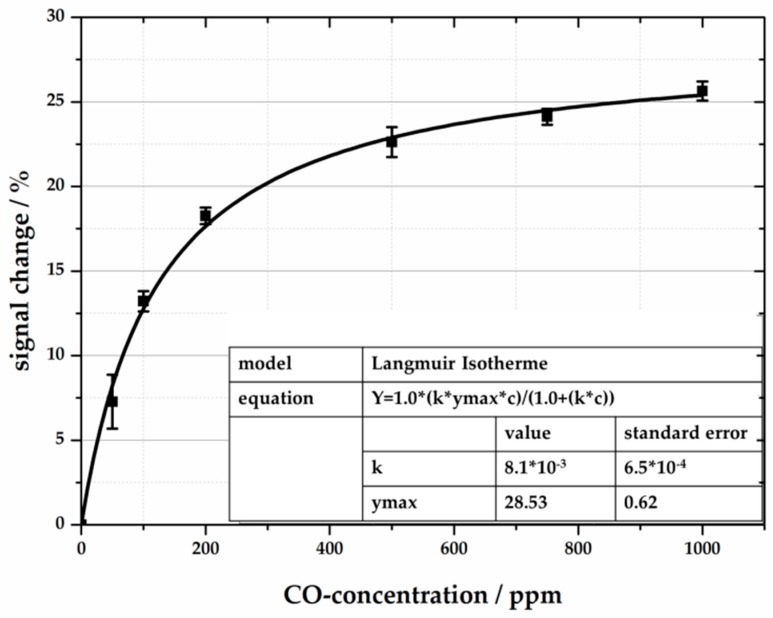
Calculated Langmuir isotherm for gasochromic CO reaction of the color dye. The reaction leads to signal changes up to 28.53%.

To characterize the CO sensitivity of our system, the reflection-based sensor is exposed to six steps of 50, 100, 200, 500, 750, and 1000 ppm CO (Figure 10). All measurements were carried out at room temperature in synthetic air and 40% relative humidity, and were repeated three times. The sensor system exhibits a quick response time of a few minutes. After stopping the CO exposure, the colored silica gel returns to its original color. The signal change correlates with the Langmuir adsorption isotherm [18], which is described in the following equation:

$$y=\frac{K_{L}\cdot y_{max}\cdot c}{1+K_{L}\cdot c}.$$

The Langmuir adsorption isotherm relates the adsorption of molecules on a solid surface in a monolayer, and is defined by the Langmuir adsorption coefficient *K**_L_***, the maximum adsorbable concentration of adsorbate *y_max_*, and the concentration of the adsorbate *c*. The Langmuir isotherm can be applied to measurements of colored silica, as it presupposes that the reaction is limited to the surface, and that all adsorption sites are equivalent. For low CO concentrations, <100 ppm CO, the output signal changes to nearly linear, whereas high CO concentrations lead to a maximum amount of substituted ligands of the binuclear rhodium complex. The sensitive layer reaches a maximum signal shift of 28.53%. Depending on the Langmuir isotherm and the derivation of the concentration dx/dy, the gradient is $4.33\frac{\mathrm{ppm}}{\%.}$. Concerning the standard error at 50 ppm of 7.27%, the detection limit can be calculated as 1σ = 6.8 ppm and 3σ = 20.1 ppm.

**Figure 11:**
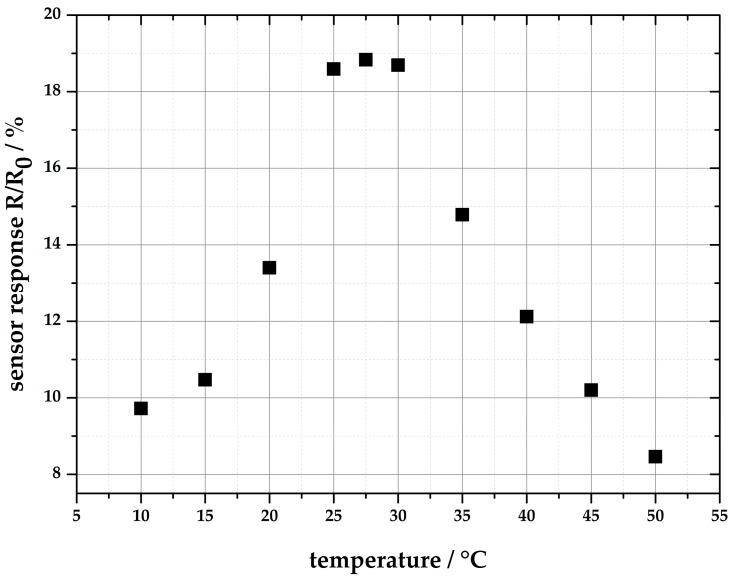
Temperature dependence of the maximum signal shift of **2∙(A)_2_** at different reaction temperatures between 10 °C and 50 °C. The colored silica was exposed to 300 ppm CO.

**Figure 12:**
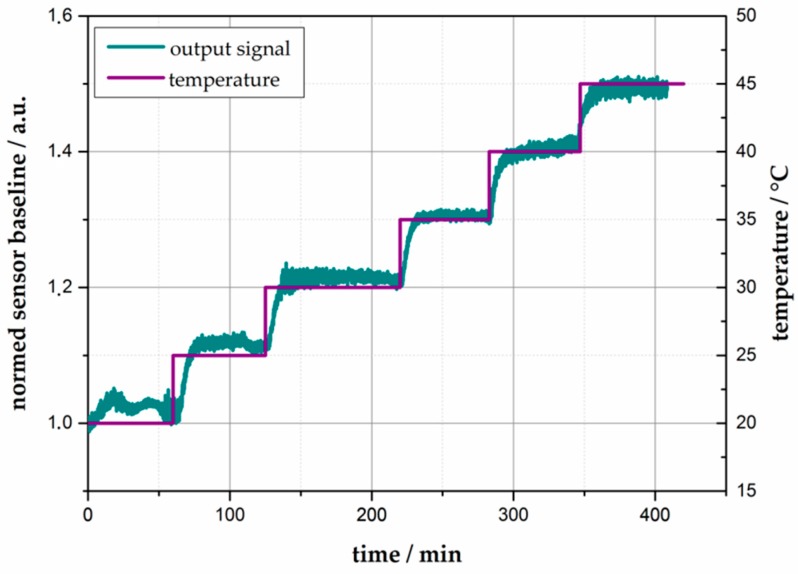
Temperature dependence of the maximum signal shift of **2∙(A)_2_** at different reaction temperatures between 10 °C and 50 °C. The colored silica was exposed to 300 ppm CO.

The influence of the ambient temperature to the CO reaction in a temperature range from 10 °C to 50 °C (in steps of 5 °C) has been investigated. Therefore, the sensor system was mounted in a climatic chamber, and the sensor was exposed to 300 ppm CO for each temperature value. The correlation between the temperature and the signal shift is shown in Figure 11. As sensor signal, the output voltage of the photo detector (R) during gas exposure is related to the output voltage at 0 ppm CO (R_0_). It was shown that the sensitivity reaches a maximum of 18.83% between 25 °C and 30 °C. At higher and lower temperatures, the sensitivity decreases rapidly. A second measurement series shows the influence of changes in the ambient temperature on the baseline of the sensor system. Again, the sensor was mounted in the climate chamber, and the temperature is increased for 5 °C every 60 min starting at 20 °C. The results are shown in Figure 12. It is obvious that the ambient temperature has a significant influence on the absorption properties of +2%/K. Both measurements were performed in synthetic air with 40% relative humidity.

A set of measurements was performed to investigate the influence of UV radiation on the dye color. Therefore, the colored silica gel (using **1∙(A)_2_**) was exposed to UV in a range from middle to UV A between 200–400 nm for 240 min. The color dye shows bleaching effects in the blue wavelength range between 450–490 nm with 3% transmission loss.

All measurements of this subsection were performed using the reflection-based measurement system shown in Figure 1b.

### 4.3. Waveguide-Based Measurements

**Figure 13:**
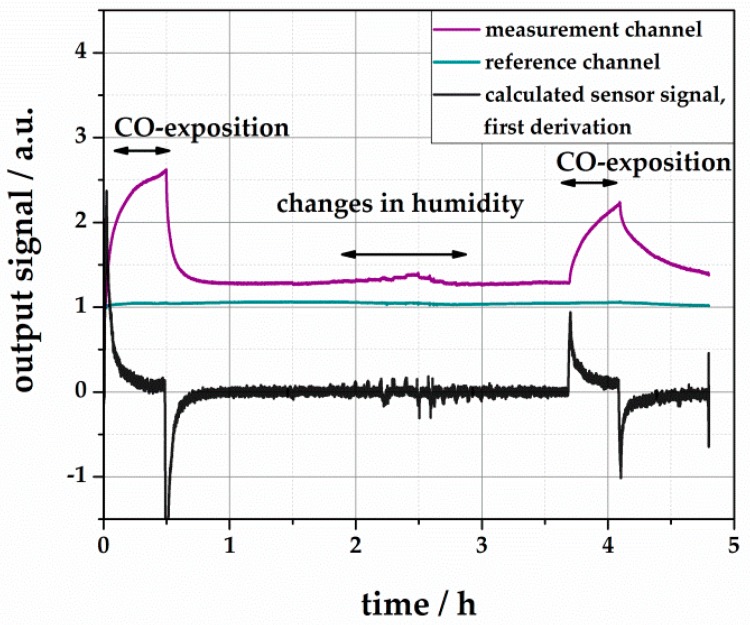
CO-dependent reaction of the waveguide-based sensor to 200 ppm CO and different background humidity. The signals are divided into the measurement and reference channel, as well as the calculated sensor signal. The measurement was carried out under ambient conditions.

A set of measurements was performed to demonstrate the fast and sensitive CO reaction of the waveguide-based sensor system. Figure 13 shows the sensor reaction to CO and a variation of the relative humidity. The signals were taken from the measurement channel and a reference channel. From that, the calculated CO sensor signal was calculated by a division. The sensor was exposed to 200 ppm CO after 30 min of stabilization. The color changes induce a signal shift of 260%. During recovery, the sensor reaches 75% after 1 h. Afterwards, the sensor was exposed to different humidity between 0% r.H. and 80% r.H. in steps of 10%. This leads to a reversible change of the sensor signal of max. 8%, which is negligible compared to the CO reactions. As the reference channel does not show any response to humidity, the signal shift is induced by the color dye and not the polymer. After another four hours, the sensor was exposed to 200 ppm CO again. The resulting signal shift is significantly lower, but still clearly detectable. The calculated response time is T_10_ = 258 s. Figure 13 shows the output of the measurement and reference channel individually. Even if the signals are stable over a few hours, it is necessary to integrate a reference channel into the measurement system, as the main sources of drifts are aging effects of the LED.

**Figure 14:**
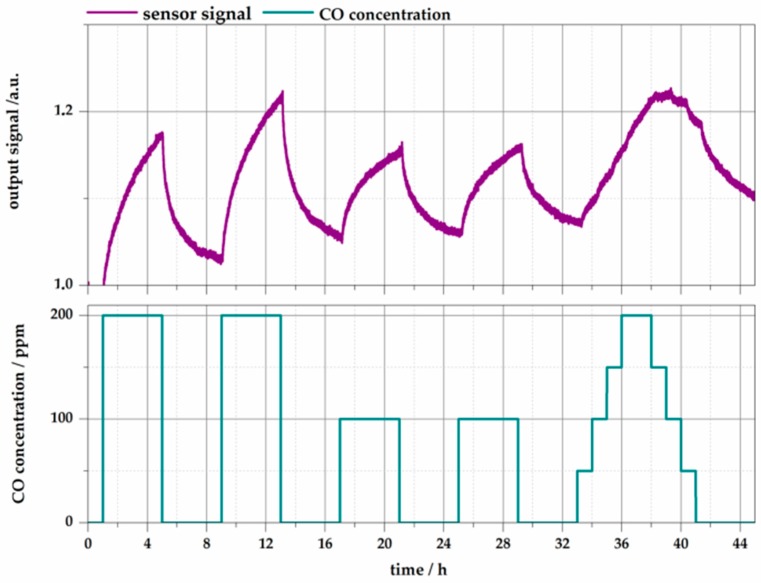
CO-dependent sensor reaction to 200 ppm and 100 ppm CO, as well as to different steps, recorded at room temperature with 40% r.H. The sensor shows sufficient repeatability over time. The measurement was performed using the sensor system shown in Figure 2b.

To demonstrate the repeatability and stability of the sensing principle, the sensor was exposed to CO for a number of times. The result is shown in Figure 14. Even after multiple CO reactions, the colorimetric change is still taking place, and reversibility is given. The measurement demonstrates the importance of the integration of a reference channel.

Another set of gas measurements was carried out, in order to evaluate the cross sensitivities to other gases, which are relevant for the targeted applications. The rhodium dye was exposed to 2000 ppm CO_2_, 50 ppm C_2_H_6_OH, 100 ppm NH_3_, and 5 ppm NO_2_ for 60 min each. **2∙(A)_2_** did not show any colorimetric reaction to these gases.

## 5. Conclusions

Five gasochromic binuclear rhodium complexes were prepared and characterized. The molecules were modified in order to improve the sensitivity towards their response to CO, by varying the ligands **A** and the residues of the phosphines **X** and carboxyl groups **R**. Each complex was analyzed by FTIR spectroscopy, ^1^H NMR spectroscopy, and differential scanning calorimetry. For all complexes, a gasochromic reaction to CO and a complete reversible behavior was observed. The color change is visible, as the dyes change their color from purple to orange, and yellow to orange. The color change was also detectable by UV/vis measurements. The modifications of ligands showed the requested influences on the sensitivity, but also on the colorfulness and reaction time. Best results were achieved by **2∙(A)_2_** and **3∙(A)_2_**, showing high sensitivity to CO and fast response time. Furthermore, the influence of the solvent on the CO sensitivity was investigated. It was shown that the solvent has a high influence on the gasochromic behavior. The insolubility in water is a main advantage for using these color dyes in sensors at ambient conditions, e.g., fire detectors. Ethanol is a suitable solvent, but suffers from long-term stability (<30 days in solution @ RT). Toluene and chloroform were made out to be useful solvents providing gasochromic reactions.

The gas sensitive complex was adsorbed on fine silica gel, and combined with LED and a photodetector for photometric measurements in reflection mode. It was shown that the reaction is sensitive, selective, and reversible. Moreover, the reaction mechanism can be characterized by Langmuir adsorption isotherm. Temperature-dependent measurements showed the significant influence of the ambient conditions to the color reaction. Realistic ambient temperatures between 25 °C and 30 °C support the ligand exchange toward **1∙(A,CO)** and **1∙(CO)_2_**. This is a positive support to obtain fast fire detection. As alternative measurement techniques, a waveguide-based sensor setup was presented. Therefore, the Rh complex was embedded into a transparent polymer matrix (e.g., ethyl cellulose) and deposited onto an optical glass waveguide. The sensor showed remarkable sensitivity towards CO, and less cross sensitivities to other environmental gases, like NO_2_, H_2_O, or NH_3_. However, based on polymer properties, this method is not completely stable at high temperatures. Moreover, color modifications in polymers require a lot of time as the reaction mechanism of the gas with the complex is limited by diffusion into the sensitive matrix. This can be proven by comparing the response times of both sensor systems. The colorimetric reaction inside the polymer is four times longer than adsorbed on a silica particle (T_10_(silica) = 80 s vs T_10_(polymer) = 258 s). The implementation of a fitting polymer is a very critical point, even in terms of longtime stability and sensor drift. As the hydrogen bonds of the substituted ligands **L** are very week, it is remarkable that the sensor shows a high repeatability, without losing the substitution partner.

The final sensor system can be improved by advanced signal processing. Therefore, a software driven lock-in amplifier can be used for reliable detection of small signal changes. The lock-in amplifier functions as a small band-pass filter. The output voltages of the photo detectors are averaged over 30 s. Within our setup, the input signal of the LED is modulated with 40 Hz. The DC output signal correlates directly with the applied CO concentration. For the application as fire detector, a fire typical critical threshold has to be defined and implemented into the microcontroller. Another important point is to control the influence of the ambient temperature to the colorimetric reaction. As the baseline of the detector signal increases with +2%/K, this value can be used for temperature compensation. As the sensor system and the gas sensitive material suffers from drift (e.g., due to temperature or humidity changes or aging effects of the LED), the implementation of a reference channel is necessary to obtain reliable CO detection. Additionally, a temperature sensor should be integrated into the measurement system. Finally, the sensor should be integrated into a dark housing to avoid direct solar radiation, and therefore, UV-induced bleaching. The main advantage using this colorimetric sensor principle as a fire detector is the strict use of low-priced components (<2 €/detector), which are, by now, already integrated in every light-scattering detector (LED, photodetector, microcontroller, alerts, etc.). As shown in [13], low-cost manufacturing, by using reel-to-reel processing, is possible. It just needs the addition of the colorimetric chip (cost of approx. 5 €/piece, depending on the used materials) to upgrade a light-scattering to a CO fire gas detector.

## Figures and Tables

**Table 1:** Ligands of the rhodium compounds **1∙(A)_2_**–**5∙(A)_2_**.

Complex	X=	R=	A=
**1∙(A)_2_**	4-OCH_3_	CH_3_	CH_3_CO_2_H
**2∙(A)_2_**	3-CH_3_	CH_3_	CH_3_CO_2_H
**3∙(A)_2_**	3-F	CH_3_	CH_3_CO_2_H
**4∙(A)_2_**	4-OCH_3_; 3,5-CH_3_	CH_3_	CH_3_CO_2_H
**5∙(A)_2_**	3-CH_3_	CF_3_	CF_3_CO_2_H

**Table 2:** Maximum transmission change (ΔT/%) of the compounds **1∙(A)_2_**–**5∙(A)_2_**, dissolved in toluene, before and after the exposure to 100 ppm CO for 10 min.

Complex	Max. Negative ΔT		Max. Positive ΔT	
	(%)	(nm)	(%)	(nm)
**1∙(A)_2_**	33.9	466	9.7	597
**2∙(A)_2_**	30.2	477	12.4	566
**3∙(A)_2_**	28.3	466	8.0	592
**4∙(A)_2_**	27.8	517	14.0	800
**5∙(A)_2_**	34.1	473	10.3	572

**Table 3:** Overview of the solubility, color, stability and CO sensitivity of **1∙(A)_2_** dissolved in five different solvents.

Solution	Solubility (c = 2.5 g/L)	Color	Sensitivity [ΔT @ 100 ppm CO]	Stability
**1∙(A)_2_** in ethanol	<1 min @RT	clear, violet	16.5%	<30 days
**1∙(A)_2_** in propane-1,2-diole	3 h @ 40 °C	clear, blue	1.4%	<60 days
**1∙(A)_2_** in toluene	<1 min @RT	clear, bright pink/violet	7.4%	for month
**1∙(A)_2_** in chloroform	<1 min @RT	clear, violet	7.8%	for month
**1∙(A)_2_** in water	not soluble			
